# River dataset as a potential fluvial transportation network for healthcare access in the Amazon region

**DOI:** 10.1038/s41597-023-02085-3

**Published:** 2023-04-06

**Authors:** Thiago Augusto Hernandes Rocha, Lincoln Luís Silva, Fan Hui Wen, Jacqueline Sachett, Anna Tupetz, Catherine Ann Staton, Wuelton Marcelo Monteiro, Joao Ricardo Nickenig Vissoci, Charles John Gerardo

**Affiliations:** 1 Department of Emergency Medicine, Duke University School of Medicine, Durham, NC 27710 United States of America; 2 Duke Global Health Institute, Duke University, Durham, NC 27710 United States of America; 3 Post-Graduation Program in Biosciences and Physiopathology, State University of Maringá, Maringá, Paraná 87020-900 Brazil; 4 Butantan Institute, São Paulo, São Paulo 05503-900 Brazil; 5 State University of Amazonas, Manaus, Amazonas 69750-000 Brazil; 6 Tropical Medicine Foundation Dr. Heitor Vieira Dourado, Manaus, Amazonas 69040-000 Brazil

**Keywords:** Health policy, Environmental impact

## Abstract

Remote areas, such as the Amazon Forest, face unique geographical challenges of transportation-based access to health services. As transportation to healthcare in most of the Amazon Forest is only possible by rivers routes, any travel time and travel distance estimation is limited by the lack of data sources containing rivers as potential transportation routes. Therefore, we developed an approach to convert the geographical representation of roads and rivers in the Amazon into a combined, interoperable, and reusable dataset. To build the dataset, we processed and combined data from three data sources: OpenStreetMap, HydroSHEDS, and GloRiC. The resulting dataset can consider distance metrics using the combination of streets and rivers as a transportation route network for the Amazon Forest. The created dataset followed the guidelines and attributes defined by OpenStreetMap to leverage its reusability and interoperability possibilities. This new data source can be used by policymakers, health authorities, and researchers to perform time-to-care analysis in the International Amazon region.

## Background & Summary

Geographic Information Systems (GIS) analysis is a powerful tool for evaluating the quality of access to medical services and acquiring fundamental data for medical decision-making^1^. In the study of neglected tropical diseases, GIS analysis is primarily used to study epidemiology and surveillance^2,3^. GIS studies involving access to care have shown that rural and remote areas remain a global concern for policymakers^4^. Topographical characteristics of remote areas, such as the Amazon Forest, create challenges to adequate access to healthcare. Additionally, addressing these challenges using data-driven solutions is difficult due to the scarcity of open-source digital geographic representations data of these locations, specifically for transportation route networks. This data gap limits the development of reliable analysis for these remote and rural regions.

While most developed countries rely on expensive GIS instruments from governmental and ordinance surveys, developing countries depend on other alternatives to overcome this limitation^5^. For example, OpenStreetMap (OSM), an open-source, volunteer geographic information mapping project, seeks to create and provide accessible worldwide geographic data on transportation route networks^6^. In the past decade, volunteers, corporations, governmental, and humanitarian organizations have developed open-source spatial data, which contributed to and used the open geographic database of OSM for various purposes^7^.

It is estimated that OSM provides an 83% complete road-based transportation route network, with more than 40% of countries having a fully mapped road network^8^. Nevertheless, data from low-income countries is uncertain, and their studies based on OSM road-based transportation route network data can have unreliable information^9^. For example, some regions, such as the Amazon Forest, have limited data availability. This region, in particular, stands out for the absence of roads, lack of regular road-based transport, and significant geographic barriers^10^. The movement of people and goods in transit between the countryside and cities is highly dependent on fluvial transportation^11–13^. In this large low-resource region, it is crucial to include rivers in developing a geographical representation of transportation route networks to better model real-world movement of people. The current inability of using rivers as a platform to perform distance analysis in the Amazon hinders appropriate data generation for research, effective disease intervention implementation, and policy-making. Any distance analysis performed in the Amazon region not taking into consideration the rivers as a pathway of transportation is doomed to be inaccurate and erroneous. Despite its importance for characterizing the Amazon Forest, to our knowledge, there is no study dedicated to developing transportation route network data sources that includes rivers as the means of people’s transportation.

The river routing literature provides methods and approaches to define hydrological connectivity, represented by the river’s connectivity network, flow direction, and surrounding terrain^14,15^. Without good methods to create a river connectivity network, the geographical representation of rivers as a means of transportation would not be possible. Our proposed database uses elements of river connectivity, but our goal was to use the geographical elements that describe river connectivity and convert them into a database structure that would be linkable to existing road-transportation networks and create vehicle-accessible paths that combined roads and rivers in the Amazon Forest. Therefore, our dataset represents how rivers can be used to create routes reflecting travel time or distance linking two points within a transportation network^16^.

Our objective was to develop a database that adapts the existing digital representation of the Amazon Forest rivers into a river-based transportation route network. To achieve this objective, we combined the river’s spatial representation with existing parameters of road-based transportation networks to allow the creation of multi-modal transportation pathways. In our database development study, we leverage previous work dedicated to studying river routing from the hydrology perspective. We combined the OSM road-based transportation network with previous existing rivers representation data freely available: the Hydrological Data and Maps Based on Shuttle Elevation Derivatives at Multiple Scales (HydroSHEDS) and the Global Rivers Classification (GloRiC)^14,17,18^.

## Methods

### Data sources

In this database development study, we combined three different data sources to develop our river- and road- transportation route network dataset applied to the Amazon Forest. Our first data source was the OSM database, which served as the primary data model for our development framework as our transportation routing notation. The OSM is the largest available source of representation of road-based transport networks. By applying the same notation to our database, we are expanding the possible uses of our dataset. The regular transportation route approaches widely applied to OSM could be replicated for this new dataset while prioritizing interoperability.

Our second data source was the HydroSHEDS database. This served as the basis for our river’s geographical representation. While countless hydrographic maps exist for well-known river basins and individual nations, there is a lack of seamless, high-quality data on large scales such as continents or the entire globe^17^. In response to these limitations, a team of scientists have developed the HydroSHEDS, a database and mapping representation of the world’s rivers that provide the research community with reliable information about rivers locations on the Earth’s surface and how water drains the landscape^19^. To create a rivers-based transportation route network dataset for the Amazon Forest rivers as the basis to perform travel time and distance analysis, we adapted the Hydrological Data and Maps Base of HydroSHEDS^14,17,19–21^. The processing steps of generating HydroSHEDS are detailed in the data set’s technical documentation^22^.

We used HydroSHEDS because it provided the best information across the geographical space of the Amazon Forest. Although HydroSHEDS has known limitations (discussed in the HydroSHEDS v.2 development website), such as low-quality resolution on some areas of the globe, it proved to be the best solution for our problem^23^. We tested the National Waters Agency (ANA) from Brazil; however, the database was composed of multiscale mapping data that provided heterogeneous resolutions of the hydrology in the area^24,25^. These resolution changes impacted the density of river streams in the analytical space, affecting the standardization of the displacement estimation. This resulted in a bias in terms of distance or travel time to access. Thus, we opted to use the HydroSHEDS data source because the river distribution is homogenous across the entire region analyzed.

In addition, the HydroSHEDS dataset has several characteristics not present in the ANA database that are similar to the road-based transportation networks, facilitating interoperability. The possibility of having lines connected by nodes to other lines creates the network structure needed to perform transportation route estimation. A transportation route estimation can be understood as an effort to estimate the best route between two or more points spatially distributed in terms of distance or time. A transportation route network dataset comprises the geographical representation of line segments (river segments) interconnected (river connectivity). Beyond the spatial representation, each line in the dataset has secondary attributes such as length, travel time, maximum supported width, direction, and maximum speed. Based on the network notion of HydroSHEDS, we were able to develop an approach to convert the geographical representation of the rivers to a transportation route network dataset.

In addition to the HydroSHEDS database, we incorporated variables from the GloRiC database, our third data source. GloRiC provides river types and sub-classifications for all river reaches contained in the HydroRIVERS database. From GloRiC, we included river characteristics such as flow regime and river stream speed. We chose GloRiC due to its compatibility with the geographical representation provided by the HydroSHEDs dataset.

### Creating the dataset

We prioritized the interoperability of our database with the community already using the OSM to perform routing analysis, facilitating the incorporation of our new dataset into existing data processing pipelines. We used the variables existing in the GeoFabrik Routable ShapeFiles from OSM. The option to use the GeoFabrik allows for additional quality checking performed over the raw version of the OSM files.

We conducted a cross-reference and compatibility assessment of the HydroSHEDS hydrographic dataset to the OSM standard. Despite the spatial representation similar to a road-based transportation network, the HydroSHEDS hydrographic dataset bears no similarity in columns informing the river code, hydrological, physio-climatic, and geomorphic data. Thus, to be able to convert the hydrologic information from HydroSHEDs into a transportation route network dataset, we had to match the OSM data notation with the available information of river characteristics in the Amazon Forest region.

The HydroSHEDS raw dataset provides data on the length of the river segment (in kilometers), but does not provide information on key features needed to calculate travel time to the closest healthcare facility. For each river segment, these features include: (a) the coordinates for the starting and ending point; (b) the estimated navigation speed for a standard boat/ship; (c) the segment access connectivity to the river network; (d) the estimated travel time through the river segment; (e) the river’s water stream flow speed; (f) seasonal variation on the flow regime; and (g) the river’s water discharge volume.

To address this issue, we created additional fields in the database to characterize the Amazon Forest rivers using information available through literature review (Supplementary Table 1). These fields can be added/modified to characterize any rivers if applicable to other areas. The Amazon Forest-specific parameters we included to translate the HydroSHEDS into a transportation route network dataset included the average navigation speed for a standard vehicle (a boat), the variations of river stream speed for a given river characteristics, the river size, and flow regime variability^26,27^. Using these parameters, we were able to calculate the average travel time to navigate through a given river segment.

Following the OSM notation, some required variables do not contribute when considering rivers as a transportation route network. Variables such as a bridge or tunnel, the number of lanes, road sizes, and presence of electricity support had no parameters meaningful for rivers. We kept the aforementioned variables in the data model to preserve OSM compatibility, but flagged as ‘do not apply’.

All the processing to adapt the HydroSHEDS rivers database to an OSM routable format was done using R Statistical Language for reproducibility and reusability. Since the spatial representation of a river’s course changes through time, we opted to build reproducible codes, allowing users to update our database to identify the best available river-based pathways. Similarly, the steps performed in this manuscript can be adapted with new geospatial databases representing the rivers of other locations.

### Transportation route database

The river-based transportation route database developed is stored and shared using the ESRI shapefile format, a widely adopted standard format to share geographical information. The majority of GIS software, including open-source solutions, are capable of reading and editing shapefiles. To create a routable dataset from the Amazon River geographic representation, we performed the following steps:

#### Definition of the origin-destination matrix to each river course

The first step defined the direction each river was flowing. This information was important to the patterns of connection of each line segment representing the rivers network. Without a proper connection standard, the data lines could not be used to solve route creation problems adequately. The rivers connect to each other differently than a network of streets. Each river connects to its tributaries at specific points. The raw dataset from the HydroSHEDS repository had a node indicating the code of how the rivers connect to each other. Despite having the code of the river segment connection, the HydroSHEDS has no information regarding the geographical coordinates of each node connection. Thus, we generated the latitude and longitude for each connection junction to be able to create the connectivity network of the river segments. This information was extracted and combined into a matrix to serve as a reference to create the rivers network. This analysis was done using R, and the code used to process this step is available on a repository^26^.

#### Implementation of transportation attributes to each river course

The second step defined the creation of the transportation attributes for each river. The transportation attributes are used to estimate travel time over a segment. These attributes include: the maximum speed allowed over a river segment; the maximum width, weight, and height allowed for a boat/ship using a specific river segment; the description of whether a river segment is accessible on foot; the estimated speed (in kilometers per hour) for a normal motorized boat/ship on this river segment; the latitude and longitude of the start and endpoints of the river segment; and the others parameters defined in Supplementary Table 1. No specific restrictions were applied for the size of the boat/ship supported by the river course, as the Amazon Forest rivers in some parts can span up to 70 km of breath. To the best of our knowledge, the only available resource to estimate travel time and maximum speed was the work performed by Moura, which indicated the average speed of a boat in the Amazon Forest^11^. Thus, we used the speed parameters presented in Moura’s study to define the average boat speed as 18.52 km per hour. According to the study performed in the Amazon region, 40% of the boats navigate at a speed of 18.52 km/h, but some boats (e.g., small boats with powerful engines) can reach up to 37 km/h^11^. In our database, we created a variable to represent the average speed and the maximum speed allowed.

Still on the second step, we added three additional variables that can be used to characterize the rivers from the GloRiC database. The variables are river stream speed, river flow regime variability, and river discharge level. The first variable is a factor that should be applied to the boat speed, as the maximum speed can change considering the navigation upstream or downstream. The riverboat velocity is the physical resultant of the boat plus or minus the river stream speed, depending on the stream navigation direction. The river flow regime can be used to identify rivers with large variability during the dry season that could affect navigability. Most parts of the Amazon rivers present a medium variability in terms of water level during the dry season. A total of 91% of the rivers in the dataset were categorized as having low (5%) or medium variability (86%) between seasons. The river discharge level variable can be used to filter rivers in terms of size, reflecting the rivers with larger chances of being navigable.

#### Labeling of parameters following the OSM standard

The other variables comprising type of surface and transportation class were set to follow the codebook associated with the OSM standard, as defined in the third analytical step^14^. We opted to use this labeling strategy to maximize the potential uses of the data generated.

**Fig. 1:**
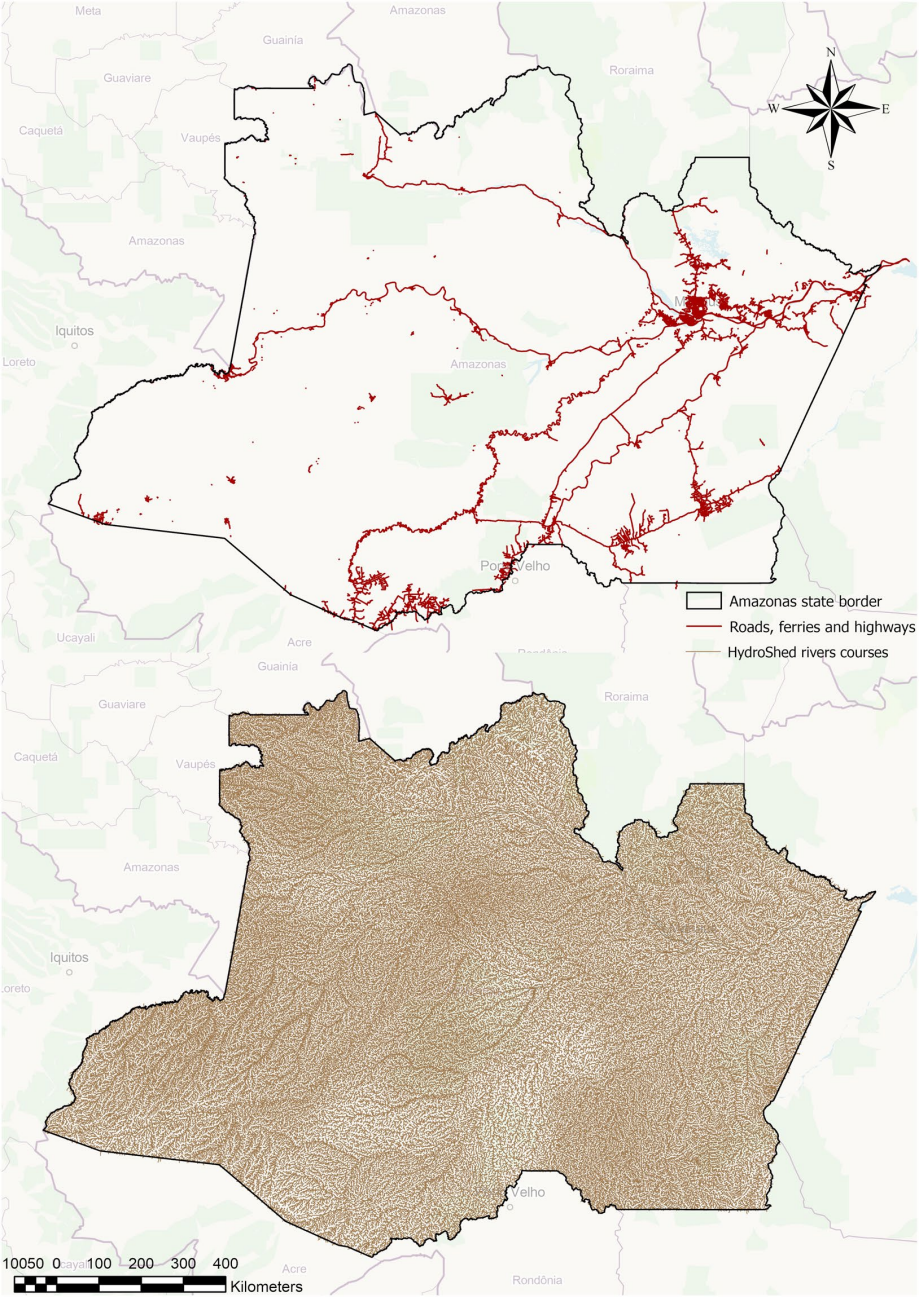
Comparison of the distribution of OSM pathways versus rivers in the Amazon Forest.

The resulting dataset from the three analytical steps is tabular data containing all the attributes of a routable dataset. To combine these attributes with the geographical representation of the rivers, we performed a linkage between the tabular data and the shapefile with the geographical representation of the Amazon Forest rivers. Figure 1 represents the distribution of OSM pathways versus rivers in the Amazon Forest region. The potential increase in the use of rivers to map distances between points can be observed by the extension of coverage of water routes in comparison to roads and highways.

**Fig. 2:**
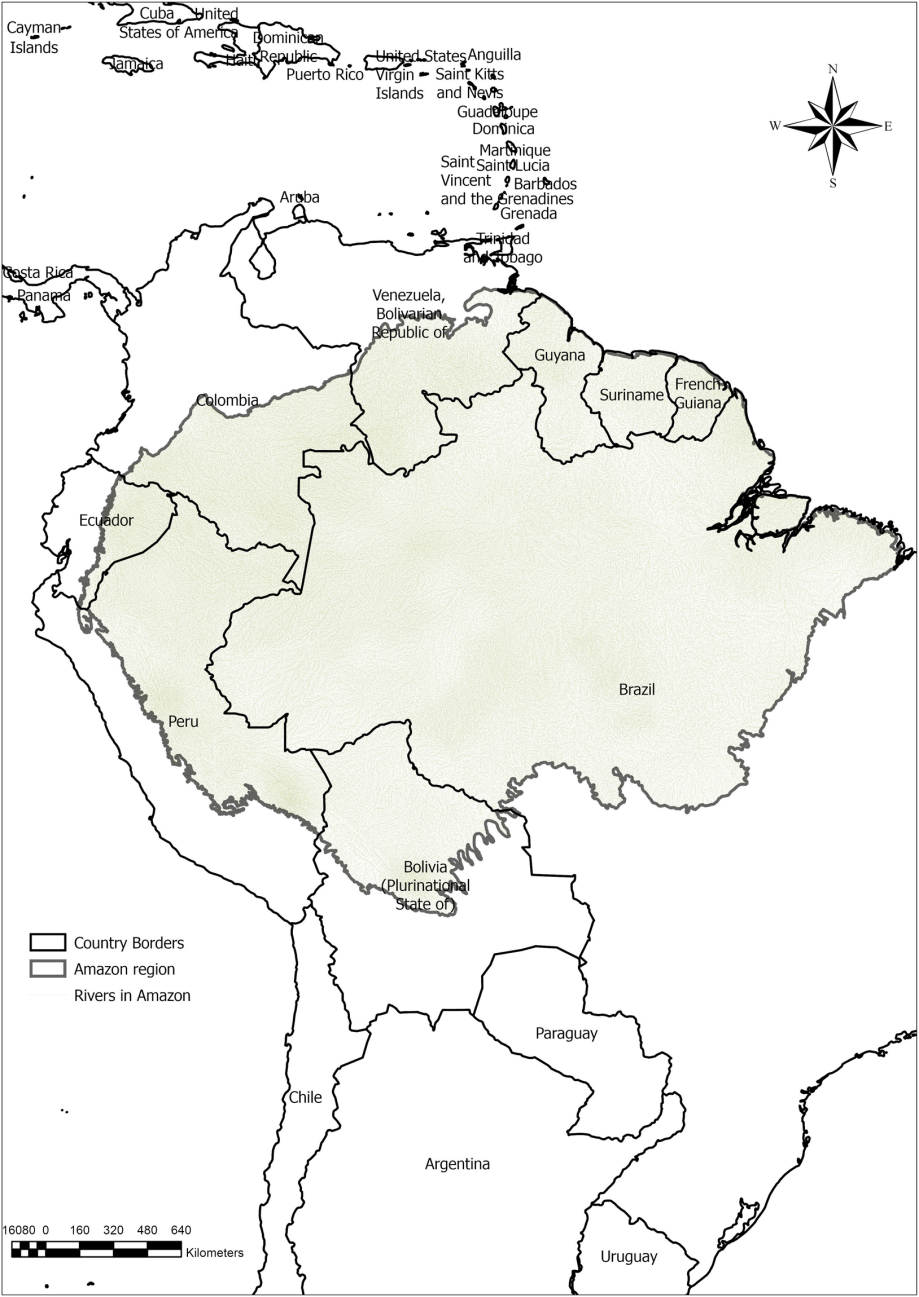
Rivers included in the routable dataset for the international Amazon Forest region.

The shapefile containing the river-based transportation route network database is stored in a repository^28^. The data source contains 897,846 river segments and 997,548 streets, roads, and highways covering the international Amazon Forest region. The river courses in Brazil, Bolivia, Peru, Ecuador, Colombia, Venezuela, Guyana, and French Guiana are covered in the new dataset, as represented in Fig. 2.

## Data Records

Here we present the metadata of our database, stored at a public repository^28^. The main file is a shapefile including metadata regarding river characteristics in the International Amazon Region. The dataset is described in Supplementary Table 1 and includes information about the size and speed of the boat, river nodes with starting and ending points, water speed, and length.

## Technical Validation

Due to the use of the new data from the Shuttle Radar Topography Mission (SRTM), the HydroSHEDs database represents the best hydrological data available.^17^No other dataset uses a Digital Elevation Model with a higher resolution and precision as the data provided by the SRTM. The NASA SRTM mission overcomes challenges in terms of data generation, surpassing limitations related to spatial resolution, data structure, multiscale approach, representation of hydrological connectivity through river networking routing, and integrated data and modeling framework^14^.

## Usage Notes

To demonstrate the potential uses associated with the new dataset, we analyzed the catchment area of a healthcare facility. Catchment area is defined as the geographic coverage using our rivers- and road- transportation route network from a set travel time distance. This type of analysis creates a catchment area by navigating the transportation network originated at the point of interest, a healthcare facility, and uses this covered area as a reference to perform calculations of the population reached, availability of health services ratio per population, rates of diseases by location, and other types of supply-demand analysis.

One application of catchment areas is to assess the population that would fall within reach of a specific health facility. Populations facing geographic barriers to access tend to present with worse health indicators in comparison to groups not facing access challenges. For example, the lack of information on where the eligible population is located represents a major issue to reach the target outcomes of health campaigns. Creating indices of access to health services often depends on data representing access routes. Inaccuracies in the access routes can lead to erroneous results in the identification of underserved areas^6,29^. The resulting misplacement of healthcare infrastructure can result in crises, such as the oxygen scarcity in Manaus at the beginning of 2021, which led to several deaths from complications of COVID-19^30^. Thus, the correct measurement of distances, travel time to reach care, and available pathways between the population and a healthcare facility are essential to assuring adequate care, better health care policies, and adequate interventions aiming to improve the organization of the health system network.

Three comparative approaches were used. In the first, we created the catchment areas close to community health centers in Amazonas state, Brazil using the road-based transportation route network dataset usually provided by GeoFabrik-OSM. The second part of the analysis was done using only the new rivers-based transportation route network database. The third analysis combined both datasets. All analyses followed the same methodology to create catchment areas using routable pathways as defined by Rocha *et al*. 2021^29^. The resulting outcome of this analysis is a geographical polygon representing the maximum threshold of distance or travel time to reach a facility considering the transportation network available. Thus, the polygon represents the coverage area of a health facility up to the limit of distance or time, defined by the end-user evaluating access. To perform the comparative analysis, we used 622 community health centers (CHC) located in the Amazonas state, Brazil as a reference for health facilities. Each CHC is responsible for offering primary health care to the surrounding population.

**Fig. 3:**
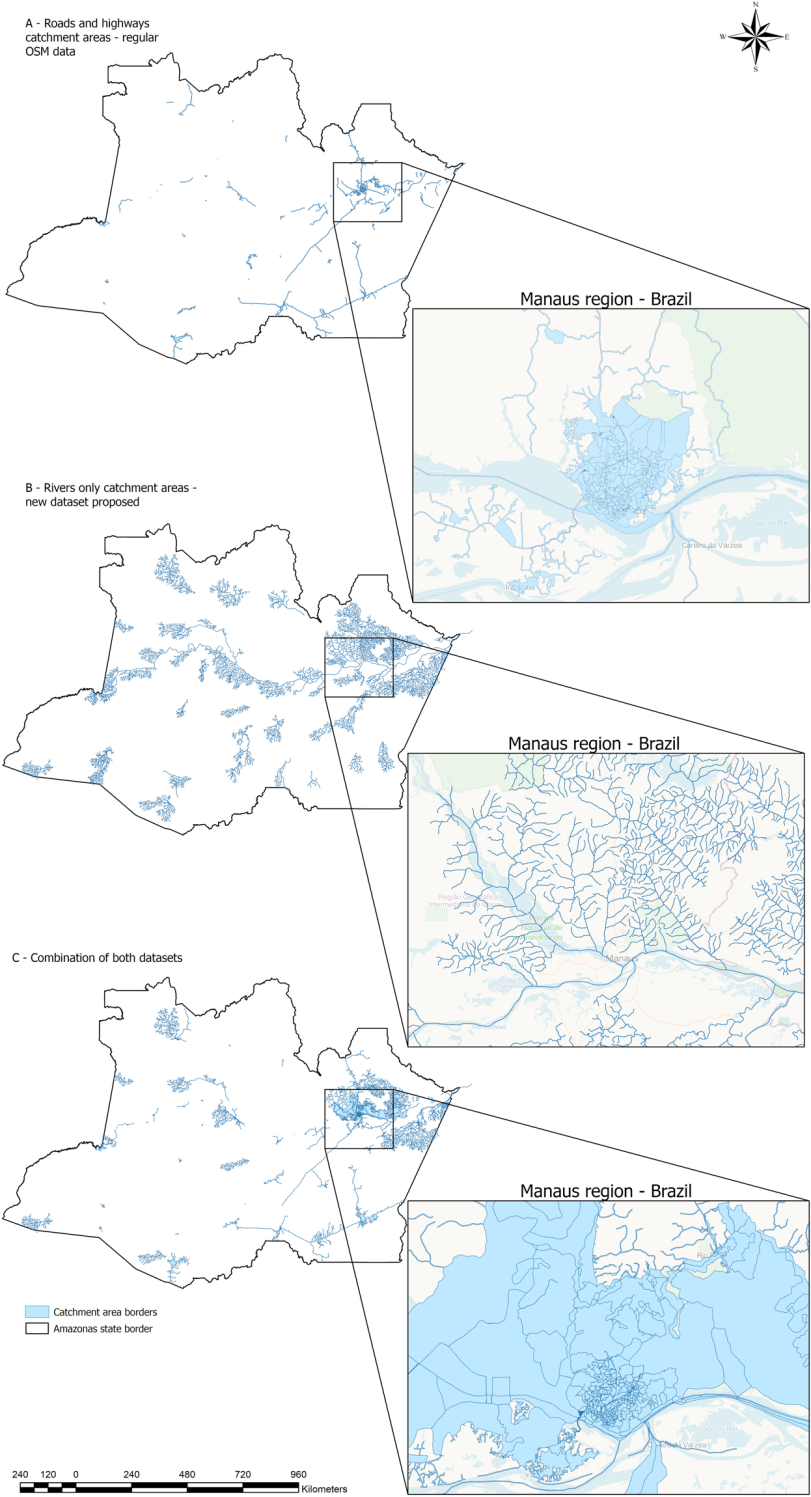
Catchment area representation using different approaches to evaluate the time to reach.

In total, we created 517 catchment areas using only roads, ferry lines, and highways as paths of access to the CHC (Fig. 3A). The catchment areas created using the new routable rivers dataset comprised 455 catchment areas (Fig. 3B). The combination of both datasets created 558 catchment areas. A total of 41 CHCs that were not reached using regular roads, ferry lines, and highways now can be included in access analysis to help health authorities better formulate policies and decisions. The use of the new dataset increases the coverage associated with health facilities by 7.5%. It is worth highlighting that the new areas covered were in remote areas not previously covered without the use of the new dataset we are proposing. Figure 3C represents an example of areas not covered by streets, but now covered by rivers as a pathway to healthcare access. Without the use of the rivers, 41 CHCs in the state would have inaccurate information on the time to reach the facility and its surrounding population.

Our results represent only the analysis of a part of the Brazilian Amazon region. We believe that the dataset presented can help several disciplines to assess more precisely time and distances in the entire Amazon region. The use of straight-line distance does not represent the actual challenges in terms of transportation in the Amazon region. A routable dataset using the rivers as pathways is an essential tool in the policy discussions regarding access to services in the Amazon region.

## Supplementary information

Supplementary Table

## Data Availability

The codes used to convert the HydroSHEDS river database to a routable dataset are freely available^28^. The code was written in R programming language, version 3.6.2. Beyond R, there is no need for any special software or program to replicate our results.
