# Supplementary material for: River dataset as a potential fluvial transportation network for healthcare access in the Amazon region
Source: Sci Data. 2023 Apr 6;10:188. doi: 10.1038/s41597-023-02085-3 (PMC10078007; doi:10.1038/s41597-023-02085-3)
Supplement: Supplementary file 1 — Supplementary Table [file 41597_2023_2085_MOESM1_ESM.docx]

**River dataset as a potential fluvial transportation network for healthcare access in the Amazon region**

Thiago Augusto Hernandes Rocha^1,2^, Lincoln Luís Silva^1,3^, Fan Hui Wen^4^, Jacqueline Sachett^5^, Anna Tupetz^1^, Catherine Ann Staton^1,2^, Wuelton Marcelo Monteiro^5,6^, Joao Ricardo Nickenig Vissoci^1,2^, Charles John Gerardo^1,2^

**Affiliations**

^1^ Division of Emergency Medicine, Department of Surgery, Duke University Medical Center, Durham, North Carolina, NC 27710, United States of America;

^2^ Duke Global Health Institute, Duke University, Durham, North Carolina, NC 27710, United States of America;

^3^ Post-Graduation Program in Biosciences and Physiopathology, State University of Maringá, Maringá, Paraná, 87020-900, Brazil;

^4^ Butantan Institute, São Paulo, São Paulo, 05503-900, Brazil;

^5^ State University of Amazonas, Manaus, Amazonas, 69750-000, Brazil;

^6^ Tropical Medicine Foundation Dr. Heitor Vieira Dourado, Manaus, Amazonas, 69040-000, Brazil.

Corresponding author: Charles John Gerardo gerar001@mc.duke.edu.

**Additional Formatting Information**

**Supplementary information**

**Catalogue**

[**Supplementary Table.S1** Supplementary Table. S1 Data format and attributes from GeoFabrik OSM routable datasets. 1](#_Toc130025451)

| **Supplementary Table.S1** Supplementary Table. S1 Data format and attributes from GeoFabrik OSM routable datasets. | | | |
| --- | --- | --- | --- |
| **Column** | **Data type** | **Description** | **Source** |
| osm_id | character | OSM ID is taken from the way identification of the feature in the OSM database. In case several features in the OSM database are joined into one feature, this is one of the Ids. This ID is not unique because one OSM object will often result in several geometry objects. This is exported as a string type since shapefiles don’t support long integers | Hydrosheds^1,2^ |
| lastchange | character | Date of last change of the OSM way | OSM^3^ and Hydrosheds^1,2^ |
| code | character | Road type code and feature class as recommended for the OSM community | OSM^3^ and Hydrosheds^1,2^ |
| name | character | Street name | OSM^3^ and Hydrosheds^1,2^ |
| ref | character | Street number (e.g. “E 20”) | OSM^3^ |
| oneway | character | Whether the street in one-way | OSM^3^ and Hydrosheds^1,2^ |
| maxspeed | number | The maximum speed, in kilometers per hour as given in the “maxspeed” tag | Combination of OSM^3^ for streets and elaborated by the authors for the rivers |
| layer | number | The layer (z-order) as given in OSM, from -5 to +5, is only used for drawing, not routing relevant | OSM^3^ and does not apply to Hydrosheds^1,2^ |
| ete | number | The estimated travel time, in seconds, on this segment | Combination of OSM^3^ for streets and elaborated by the authors for the rivers |
| speed | number | The estimated speed, in kilometers per hour, for a normal motor vehicle (or boat/ship) on this segment, derived from the road type and adjusted by the max speed value, if any | Combination of OSM^3^ for streets and elaborated by the authors for the rivers |
| length | number | The length of this segment in meters | OSM^3^ and Hydrosheds^1,2^ |
| bridge | character | Whether this segment is on a bridge, ‘T’ for true, ‘F’ for false | OSM^3^ and does not apply to Hydrosheds^1,2^ |
| tunnel | character | Whether this segment is in a tunnel, ‘T’ for true, ‘F’ for false | OSM^3^ and does not apply to Hydrosheds^1,2^ |
| maxwidth | number | Maximum vehicle width in meters | OSM^3^ and does not apply to Hydrosheds^1,2^ |
| maxheight | number | Maximum vehicle height in meters | OSM^3^ and does not apply to Hydrosheds^1,2^ |
| maxweight | number | Maximum vehicle weight in tons (1,000 kilograms) | OSM^3^ and does not apply to Hydrosheds^1,2^ |
| surface | character | The surface property specified in OSM. Frequent values are “asphalt”, “paved”, “unpaved”, “ground”, “gravel”, and “concrete”; see<https://taginfo.openstreetmap.org/keys/surface#values> for details | OSM^3^ and does not apply to Hydrosheds^1,2^ |
| lanes | number | Number of lanes (sum of both directions) | OSM^3^ and does not apply to Hydrosheds^1,2^ |
| edge_id | character | Unique ID of this segment (edge), starting at 1 for the first edge in the file. Note that this ID is not constant across deliveries, i.e., a later similar export will have different IDs. | OSM^3^ and Hydrosheds^1,2^ |
| start_lat | number | Latitude and longitude of the start and endpoints of this segment. Adding these columns unnecessarily duplicates information already contained in the geometry but may make some forms of processing easier | Combination of OSM^3^ for streets and elaborated by the authors for the rivers |
| start_lon |  |  | Combination of OSM^3^ for streets and elaborated by the authors for the rivers |
| End_lat |  |  | Combination of OSM^3^ for streets and elaborated by the authors for the rivers |
| End_long |  |  | Combination of OSM^3^ for streets and elaborated by the authors for the rivers |
| start_node | character | OSM node IDs of the start and endpoints of this segment (may be used in routing graph construction—same ID means there is a link). These fields are strings, not integers, because the shapefile format does not support long integers. Node IDs in OSM do not fit into an unsigned 32-bit integer | Combination of OSM^3^ for streets and elaborated by the authors for the rivers |
| end_node |  |  | Combination of OSM^3^ for streets and elaborated by the authors for the rivers |
| motorroad | character | Whether this road segment is a motorroad (directly derived from the OSM tag motorroad=yes/no). Uses the values “f” for false and “T” for true. This field is usually not set for motorways because they imply motorroad=yes | OSM^3^ and does not apply to Hydrosheds^1,2^ |
| a_foot | character | Whether this road segment is accessible to pedestrians, cyclists, motorists, heavy goods vehicles (truck/lorries), or public service vehicles (buses). Uses the values “F” for false, “T” for true, “P” for private (same as “F” for most purposes”), and “D” for delivery/destination (meaning this mode of transport is only allowed if going to or coming from an address in the immediate vicinity) | OSM^3^ and does not apply to Hydrosheds^1,2^ |
| a_cycle |  |  |  |
| a_motor |  |  |  |
| a_hgv |  |  |  |
| a_psv |  |  |  |
| dowstream | numeric | Stream velocity to be added to the boat/ship speed when transiting downstream in the segment | GloRiC^4,5^ |
| upstream_s | numeric | Stream velocity to be deducted from the boat/ship speed when transiting downstream in the segment | GloRiC^4,5^ |
| regim | character | Flow regimen variability of the river segment in terms of water level | GloRiC^4,5^ |
| discharge | character | Categorization of the river size from the volume of water discharged | GloRiC^4,5^ |

**References**

1. Lehner, B., Verdin, K. & Jarvis, A. HydroSHEDS technical documentation (available at http://hydrosheds.cr.usgs.gov). *River Res. Appl.* (2006).

2. Lehner, B. et al. HydroSHEDS v2.0 &ndash; Refined global river network and catchment delineations from TanDEM-X elevation data. (2021). doi:10.5194/egusphere-egu21-9277.

3. Kloog, I., Kaufman, L. I. & de Hoogh, K. Using open street map data in environmental exposure assessment studies: Eastern Massachusetts, Bern region, and South Israel as a case study. *Int. J. Environ. Res. Public Health* (2018). doi:10.3390/ijerph15112443

4. Lehner, B. & Grill, G. Global river hydrography and network routing: Baseline data and new approaches to study the world’s large river systems. *Hydrol. Process.* (2013). doi:10.1002/hyp.9740

5. Lehner, B., Verdin, K. & Jarvis, A. New global hydrography derived from spaceborne elevation data. *Eos (Washington. DC).* (2008). doi:10.1029/2008EO100001
